# The role of cultural values and norms in the adoption and implementation of foreign innovations in health service delivery in China

**DOI:** 10.3389/frhs.2025.1401641

**Published:** 2025-07-18

**Authors:** Wenxing Wang, Jeroen van Wijngaarden, Martina Buljac-Samardžić, Joris van de Klundert

**Affiliations:** ^1^ Health Services Management & Organization, Erasmus School of Health Policy & Management, Rotterdam, Netherlands; ^2^ School of Business, Universidad Adolfo Ibanez, Santiago de Chile, Chile

**Keywords:** cross-culture, China, innovations, health services, implementation, cultural norms and values

## Abstract

**Background:**

In pursuit of the ambitious and large scale 2009 national health reform objectives, China has turned to the introduction and implementation of foreign best practices in health service delivery. While it is well known that cultural differences may significantly impact the adoption and implementation of innovations, there is little specific knowledge on how these impact foreign health service innovations adopted in China. Our aim is therefore to identify which cultural norms and values affect the adoption and implementation of foreign innovations in health service delivery in the Chinese context and how.

**Methods:**

We interviewed a variety of respondents (government officials, directors, doctors, consultants, researchers) involved in the adoption and implementation of foreign health service delivery organizations from China (*n* = 10) and from The Netherlands (*n* = 9), with which China has built long-standing cooperation on exchanging innovations in health service delivery. A semi-structured interview guide was used. The interviews were transcribed verbatim, translated (Chinese into English) and coded, using both thematic and open codes.

**Results:**

Identified values and norms related to health and care, health services and professionals, and organizational dynamics respectively. Some act as potential barriers to the adoption of innovations (e.g., the body must be treated with respect), some play a facilitating role (e.g., eagerness to learn from other cultures), and some can be either a facilitator or a barrier depending on the circumstances or the views of our respondents (e.g., filial piety, longevity is valued over quality of life).

**Conclusions:**

Innovations in primary and elderly care that are nurse-led and focus on new service concepts emphasizing personalized care, experience cultural barriers in China. Meanwhile more structural and technical innovations especially in a hospital setting are strongly facilitated by Chinese cultural characteristics. While the national culture has a significant impact on foreign innovations in China, the interactions of those innovations with the local cultural context at meso- and micro- levels additionally plays an important role.

## 1 Introduction

The Chinese government initiated its national health reform in 2009 to establish an accessible, equitable, affordable and efficient health system (1). A major priority has been to improve health service delivery (2). China therefore has called for innovations of health service delivery and has pursued the implementation of foreign innovations (3). These innovations have often been developed in countries with essentially different national and organizational cultures. Compared to many of the countries from which these innovations originate, China for example has a higher power distance and a more collectivistic society (4). Such cultural characteristics can have a significant impact on the uptake and implementation of innovations (5, 6). Moreover, the impact of cultural factors may be particularly pronounced as the differences with the country of origin of the health service delivery grows larger, as is for instance between China and The Netherlands (7).

Culture is defined by Spencer-Oatey as “a fuzzy set of basic assumptions and values, orientations to life, beliefs, policies, procedures and behavioral conventions that are shared by a group of people, and that influence (but do not determine) each member's behavior” (8). Within studies on implementation science, culture has been hierarchically organized as national (macro level, cross-cultural), organizational, group (sub-culture, professional, special interest, social class, etc.) and individual culture (micro level, subjective culture) (9). All individuals are encircled by a cultural web of norms and values, which is an important foundation for communication and interaction (9). Cultural and social mechanisms on each of these levels influence behavior and therefore may open “doors” or form “walls” for innovations from outside this cultural context (9).

Several studies have focused on the general effect of culture on adoption and implementation of innovations and on a country's innovativeness, mainly based on Hofstede's model of cultural dimensions (see Supplementary Table S1 in Supplementary Materials) (5, 6, 10, 11). For example, high scores on the dimensions power distance, collectivism, and masculinity have been reported to positively impact adoption and implementation of innovations (5, 6). At the same time, high scores on long-term orientation seem to positively impact adaptation and implementation of innovations (5, 6). For example, China's low scores on uncertainty avoidance and high scores on long-term orientation seem to positively impact adaptation and implementation of innovations. However, few studies have considered the specific effects of cultural differences between the country in which the innovation is adopted and implemented with the country from which the innovation originates. Some Western innovations may poorly transfer to non-Western contexts, such as the Asian continent because of these differences (12). For example, the Netherlands has a much more individualistic culture compared to China, which may be incorporated in the Dutch health service models that are being adopted in China (5, 6).

When addressed, cultural factors are mostly considered as one of several categories of factors and primarily for specific innovations (10, 11). For instance, Zhou et al. (10), reported China to encounter more difficulty with implementation of palliative care for people with heart failure because of the value attached to longevity in Chinese thanatology. Ye et al., reported that China's centralized health system faced challenges in decentralizing tasks among health organizations when introducing telemedicine, especially during the COVID-19 pandemic (13). Jiang et al's study also showed that Chinese residents have a preference for tertiary hospital care over community care (14).

A focused in-depth analysis of the role cultural differences play in the adoption and implementation of foreign innovation in health service delivery by China is lacking, despite their importance in the Chinese national health reforms. Therefore, current understanding of how culture influences China's introduction and implementation of health service delivery innovations from abroad remains scattered. To advance and structure understanding of this matter, we conducted a qualitative study guided by the following research question: which cultural norms and values affect the adoption and implementation of foreign innovations in health service delivery in the Chinese context and how? This research may contribute not only to understanding the cultural impact on innovation adoption within the Chinese context but also to offering broader insights into how cultural factors affect the adoption of foreign health service innovations across diverse contexts with significant cultural differences.

## 2 Methods and materials

### 2.1 Data collection

This study is part of a larger study which aims to generally identify which factors influence the uptake and implementation of foreign innovations in health service delivery in China and how. The larger study has adopted a qualitative design (15), because it permits the in-depth exploration of factors, among which are the cultural factors, and how the factors exert influence.

The interview protocol of the general study was developed based on Greenhalgh's conceptual model which considers multiple determinants for diffusion, dissemination, and implementation of innovations in health service delivery and organization (16). This manuscript is confined to the cultural factors (specifically cultural values and norms) and how they impact the aforementioned adoption and implementation. The questionnaire consisted of open-ended questions for probing in-depth information and were pilot tested in the first several interviews. We used the different levels as identified by Greenhalgh, to probe for relevant values that influence the uptake of innovations, namely: the general Chinese context, the health system, the health care organizations and professionals, the users, the type of innovations, values related to resources and finally values related to implementation processes (16).

All interviews were conducted online via We-chat calls between March and May 2021. The interviews lasted between 35 and 78 min (mean 60 min). The first author audio-recorded, transcribed all interviews, and translated Chinese interview transcripts into English (17).

### 2.2 Participants

As reflected in the nature of the research, views on the factors influencing the uptake of foreign health service delivery innovations may vary as differences in cultural norms and values yield different perspectives on the uptake and on how it is impacted by cultural factors. Hence, we purposely selected Chinese and foreign experts. Chinese respondents may have a deeper understanding of the actual implementation and the Chinese culture. Foreign experts may better understand the original innovation and their outside view may reveal factors at work which are difficult to observe by Chinese experts.

Such differences in views will be most pronounced when involving foreign experts from a cultural background which differs considerably and essentially from the Chinese on important dimensions, such as power distance and collectivism (4). Moreover, it is beneficial to involve participants from foreign contexts from which a variety of best practices have been introduced to innovate health service delivery in China, to ensure respondents draw from a rich set of experiences.

The Dutch-Sino cooperation on health care has a long-standing history of over 20 years (15). China entered this cooperation because of the widely recognized quality of the Dutch health system, and specifically because of its advancements in primary care and elderly care, which form important priorities in the Chinese health reforms (2, 18). As The Netherlands also scores essentially different from China on most dimensions of Hofstede's organizational culture model, it forms a valuable context for the selection of foreign experts (19). We have therefore selected Dutch respondents which have been involved in introducing Dutch innovations as part of the Dutch-Sino cooperation.

In total, we were able to recruit 19 respondents that met our criteria, using our Chinese contacts, and via a snowballing technique including 10 Chinese and 9 Dutch respondents. All participants were deliberately selected based on their expert knowledge and experience in implementing innovation projects in the Chinese context. The sample of Chinese respondents was generated with the help of the Health Human Resources Development Center, Ministry of Health of the People's Republic of China. Meanwhile Dutch respondents were recruited using a snowballing technique, via the fourth author's network with the Ministry of Health, Welfare and Sports of the Kingdom of the Netherlands, and with the WHO. To mitigate selection bias, we established clear criteria for participant selection to ensure diversity in respondents' backgrounds and affiliations. This included representation from different organizational levels, such as primary community care, tertiary hospitals, governmental bodies, and academic institutions. Also, participants should have at least 5 years of experience with the implementation of foreign innovations in China.

Experts were from a variety of affiliations, including healthcare governmental departments (as governmental officials), provider organizations (as directors, administrators, doctors), universities (as researchers), and consulting organizations (as innovation project collaborators).

### 2.3 Data analysis

A thematic content analysis was conducted in Atlas-ti software and Microsoft Excel, to identify and generate patterns (17). The coding process was inductive (20). The first and second author independently analyzed and coded transcripts, with a bottom-up approach, by moving between phrases recommended in practice guideline (20). For example, “CT and “x-ray” were coded as “hard skill” while “medical consultancy”, “empathic to patients” were coded as ’soft skill.” During the analysis process, transcripts were familiarized, initial codes formed, themes searched and reviewed, themes defined and named, and then report produced. With the completion of initial coding, the dissimilarities of codes labelled by the first and second author were compared and discussed until consensus was reached. For instance, after discussion, the analytical view changed, and tech labels for “CT” and “x-ray” changed from “hard skill” to “tangibles” while “soft skill” was changed to “intangibles”. Then themes were differentiated or merged into overarching themes, and categories were generated. For instance, the category of “values and norms related to health services and professionals” emerged by combining several themes such as “tangibles valued over intangibles”, “health care as public service vs. economic transaction.”

The credibility and reliability were assured by consensus review and appraisal of themes from transcripts among all authors. To optimize cultural sensitivity, interview questions were designed with attention to culturally relevant language and context. Knowledge from both cultures was represented within the respondent group and in the research team. Both consisting of Dutch and Chinese citizens. Part of the data-analyses was also a continuous reflection within the research team on possible cultural biases. Pseudonyms were used throughout to protect participants' anonymity.

### 2.4 Ethical issues/statement

This study is not subject to the Medical Research Involving Humans Subject Act and has been approved by the Research Ethics Review Committee of the Erasmus School of Health Policy & Management on 19th February 2021 (reference: 21-002). All respondents have verbally provided informed consent on record, based on a standardized consent form that was adapted for this study and send to them beforehand. Part of the ethical procedure was a privacy and data-management check. As the interview data could not be anonymized but only pseudomized, primary data will not be shared with other researchers and are safely stored.

## 3 Results

Three categories of cultural values and norms influencing the uptake and implementation of foreign innovations in the Chinese context were generated from our data analysis. First, values and norms related to health and care. These refer to values and norms influencing people's expectations and preferences on health and care. Second, values and norms related to health services and professionals. These refer to values and norms influencing peoples' perceptions towards and choices on health services, professionals, and healthcare settings. Third, values and norms related to organizational dynamics. These refer to values and norms influencing peoples' behaviors, learning styles in a workplace and patterns of interactions between people within and outside the organization.

### 3.1 Values and norms related to health and care

Within this category, four specific values were discussed by our respondents. Two of these values relate to filial piety, which is a shared attitude of respect for parents. It includes the expectation that children provide services to their parents and the traditional value of “the body must be treated with respect”. The other two values are the appreciation of Traditional Chinese Medicine and valuing longevity over quality of life.

#### 3.1.1 Expectations of family caregiving

Filial piety (also known as Xiao) is a commonly shared attitude of respect for parents in China, which requires children to provide services for their parents including social, material or daily life support (21). According to our respondents this cultural value can both be a barrier and a facilitator for the adoption and implementation of innovations from abroad, especially regarding innovations related to elderly care.

Several respondents mention that it is unacceptable in some regions in China, such as the Chaoshan area where the Hakka culture plays an important role, to outsource the task of providing services to one's parents to non-family members. Consequently, innovations such as institutional and community-based elderly care encounter a barrier.

“Unconsciously, they think if you go to those day care centers, that means your children are neglecting filial piety. I don’t know if this tradition has influence in many areas, but regions like Chaoshan, Guangzhou with the Hakka culture have an old tradition of supporting the elderly parents as the children/family's responsibilities. So, day care centers are not well utilized.”

Different respondents, however, mentioned that filial piety may also facilitate the implementation of service innovations relevant to the elderly care, for example in developed cities like Shanghai. Younger generations are often occupied by their work. Their incomes have increased but they often lack the time to take care of their parents. Innovations in elderly care services, such as intensive homecare and new forms of elderly institutions, allow younger people to assume their responsibilities and obligations to their parents (filial piety) by outsourced and paid caregiving. They cover (part of) the costs which the elderly themselves cannot afford.

“And because of the current development of society. In developed cities like Shanghai, children have their own careers and have to raise their own children, so a lot of energy can't be used to take care of their parents.”

“Middle-aged people are more stressed in making money and can't take care of their parents. The elderly institutions would be a relatively important industry in the future.”

#### 3.1.2 The body must be treated with respect

As written in the “The Classic of Filial Piety”, “The body, our hair and skin are all given to us by our parents. Not daring to damage any part of the body is the beginning of filial piety.” (22). That means it is disrespectful to your parents to damage your body. Various authors use this value to explain the underdevelopment of anatomy in the Chinese medicine and the resistance to giving body parts to others (e.g., blood, organ donation) (22). A Dutch respondent gave an example of how difficult it can be to implement evidence-based medicine and practices which require regular collection of participants' blood samples. This required additional efforts from health professionals, including clear, patient-centered explanations, involving family members in discussions to build trust, and using educational materials to reframe blood donation as an act of safeguarding health rather than as disrespectful to parents.

“That was what I was told, in China people are hesitant to come to the hospital and give blood. Because that’s part of their body, which comes from their parents. So, you have to be careful with this. So it was quite difficult to convince them that we must have so many, so frequent blood collection. This is kind of in the culture.”

#### 3.1.3 The value of traditional Chinese medicine (TCM)

TCM is well embedded in China (23). TCM treatments come in various forms including herbal medicine, acupuncture, cupping therapy, gua sha, massage (tui na), bonesetter (die da), exercise (qi gong), and dietary therapy (24). Those treatments are used for many different afflictions from chronic pain to infertility. But TCM is not only regarded as a treatment according to our respondents, it also embodies Chinese traditions, which people think are important to pass on.

“I know a lot of Chinese, especially the elderly, are fond of Chinese alternative medicine. For example, to solve sleeping problems, or if you have a cold, or if you have muscle pain.”

“Meanwhile our traditional culture of Chinese medicine should also be carried forward. Because culture has to be passed down and to be developed.”

There are different opinions between our Dutch and Chinese respondents on whether TCM popularity has a negative or positive impact on the implementation of foreign innovations. It is interesting to note in the quote above that the Dutch respondents classify it as “alternative”, where the Chinese name it “traditional”. In the Dutch context, “alternative” officially refers to medical practices that are not part of standard medical care but is also often used to refer to treatments which are not considered to adhere to the recent standard of evidence based medicine. While “traditional” in the Chinese context refers to treatments rooted in tradition and based on ancient philosophical concepts (25). These contextual differences in views and values may also explain why some, mostly Dutch, respondents perceive the focus on existing believes and tradition to stand in the way of innovation in Chinese health care.

“Culture problems, there are too many people who still believe that if you won’t eat a carrot, you will get a cancer; and if you have cancer, you have to eat more carrots, then it will go away. People only believe what they want to believe. That’s the problem in China and less in Holland, but it’s still there. You have to really talk to people, to explain why you want the innovation and what’s the benefit of it.”

However, according to our Chinese respondents, people in China do not always refuse innovations such as evidence-based medicine and practices due to TCM belief. They are open for other treatments and decide for themselves under which circumstances they use TCM, Western Medicine or a combination of both. TCM is especially used to improve general health and relieve physical discomfort with chronic diseases (25). The use of TCM is a cultural tradition they want to keep alive according to our respondents. So, they look for ways to integrate different types of treatments. For example, according to a Chinese respondent with a professional background of TCM, health professionals are fully open to integrative medicine practices which combines Western Medicine and TCM. Therefore, according to our Chinese respondents the popularity of TCM in China does not necessarily have a negative impact on implementing foreign innovations, it could even play a facilitating role.

“I think there is no conflict, it should be an organic combination. Because I studied Chinese medicine, and I spent 3 years in the rehabilitation department. I think that Chinese and Western medicine is not mutually exclusive, but should be combined with each other. Chinese medicine could be a factor, I think, in the implementation of foreign innovations. Because traditional Chinese medicine is popular and of its own characteristic. But it can be compatible with Western medicine, and complementary. Some of the advanced ideas in the West are worthy of our learning. Meanwhile our traditional culture of Chinese medicine should also be carried forward. Because culture has to be passed down and to be developed.”

#### 3.1.4 Longevity is valued over quality of life

Longevity is valued over quality of life in China, according to several respondents. This value can facilitate or inhibit the implementation of innovations from abroad. Several respondents state that Chinese families try everything to prolong their significant one's survival, regardless of medical expenses, disease prognosis and expected quality of life. This can facilitate the implementation of technological innovations especially in hospitals, such as proton therapy. However, it may also impede innovations such as hospice care.

“In the dimension of unrealistic concepts of life and death, it leads to the rapid application and even abuse of many innovative technologies in hospitals. That’s my opinion.”

“Everybody will die eventually. But still, people want their families to be treated by the best technologies, even if the family has no money, they still want to use.”

### 3.2 Values and norms related to health services and professionals

Within this category, four specific values were discussed by our respondents namely valuing tangibles over intangibles, healthcare as public service vs. economic transaction, lack of appreciation of and trust in primary care health professionals and services, and the relatively low appreciation for nurses.

#### 3.2.1 Tangibles valued over intangibles

Various respondents stated that in China tangibles such as facilities and equipment are valued over intangibles, such as communication and empathy, which are more service-related and often difficult to measure. The emphasis on tangibles can form a barrier for the adoption and implementation of service innovations such as homecare services, while being a facilitator for technological innovations. Our foreign respondents gave examples of how difficult it was to get support for innovations in elderly care that did not involve the use of fancy new equipment or modern buildings:

“But they don’t understand the value of intangibles. They only focus on how many facilities (e.g., care centres in communities) you have, and whether you have high-end equipment. He doesn’t understand that the elderly do not need those things. But you have to show leaders that your organization is equipped with those high-tech equipment. Leaders want you to present hardware, nice buildings for example. They don’t understand the core of community care. Attention should be paid to how clients feel about care provided.”

These preferences are also reflected in payment schemes of health professionals according to our Chinese respondents. For example, a doctor receives only 30 RMB per consultation, while a CT scan will bring in 250 RMB and an MRI scan brings in 800 RMB. Investing in intangibles may therefore not increase earnings that can cover the investment (or even reduce earnings). This has demotivated and even frustrated Chinese professionals investing in intangible innovations.

“It’s definitely a pricing problem, and that’s what now gets in the way of these innovations, without a good reflection of the value of people providing services. The pricing system is a good reflection of the value of things (medical products, tangibles) instead of the value of humans, right? The underpayment of health workers has not been solved in China, because Chinese people do not recognize/ appreciate the value of service provision. They would like to pay for medicine administrated, medical examinations via equipment, but our consultation fee is cheap, just 30 RMB. You say, how much would it cost if you want to see an expert clinician in Europe?”

#### 3.2.2 Health care as public service vs. economic transaction

According to some (and only) Chinese respondents, health professionals in China regard health service provision as a public service; a commitment to societal well-being. However, according to those respondents, patients in China often regard health service provision purely as an economic transaction, during which they (buyers) purchase services from health providers (sellers) and expect value for money.

“I think healthcare cannot be regarded as a service industry. It is about empathy for people who need medical help. We can't use the title of ’service’, because I'm not, serving but helping you. I think it’s better to call it as medical help.”

“You’re the seller, he’s the buyer. He’s buying and paying for your services, and then you're selling technology and giving him something to keep him safe, without the feeling of being helped by a kind, admirable health practitioners.”

As “buyers” Chinese patients often do not accept unexpected or unwanted outcomes of health services. They expect value for money, perhaps because high out-of-pocket medical expenses often result in a considerable financial setback for a family. If the outcome of a treatment is not satisfying, conflicts with health professionals can easily arise. This makes Chinese health professionals reluctant to take risks, which may act as a barrier for adopting innovations such as innovative surgery methods.

“A selling and buying culture related to healthcare and medical treatment; So the risk of innovations cannot be accepted; Doctors often take patients’ financial capabilities and tolerance to unpredictable medical outcomes into consideration, so as to avoid unwanted consequences (e.g., lawsuit, doctor-patient conflicts).”

#### 3.2.3 Undervalued and distrusted health professionals and services in primary care

Various Dutch and Chinese respondents mentioned that primary care professionals and services are undervalued and distrusted in China. Chinese citizens primarily value hospital care and mostly from higher level hospitals (e.g., tertiary hospitals), especially so in economically developed cities such as Beijing, Shanghai and Guangzhou. Patients often skip local primary healthcare institutions and directly turn to tertiary hospitals for health services, while health professionals themselves prefer employment by secondary and tertiary hospitals over working for primary care institutions. As a consequence, innovations especially focused on primary care services have encountered barriers such as mistrust and inadequate human resources. According to our respondents from both countries:

“The whole society does not trust the basic health care system, because they get used to go to the tertiary hospitals.” (Chinese)

“But deeply rooted ideas still dominate, and they have doubts about the professional competence of community caregivers.” (Chinese)

“Indeed, health practitioners, nurses are more willing to working in the tertiary hospital.” (Chinese)

An example given by our respondents is the introduction of general practitioner services (also known as family doctor services):

“There are particularly few paid service provisions, because people don't see, don't understand, don't think family doctor is something of value.” (Dutch)

“They will directly go to large hospitals for medical services, even if they have to pay medical expenses, instead of going to the primary health institutions with medical insurance compensations.” (Dutch)

However, it seems that foreign innovations in primary care are better implemented in rural areas than in urban regions. Our Chinese respondents gave different explanations. Some believe that interpersonal relationships are closer and more trustworthy in rural areas. It is more likely that health practitioners and patients are acquaintances.

“Well… I think people in rural areas are easier than those in urban areas to trust township and village hospitals. Villagers naturally have a close bond with their village doctors, with their local hospitals, so they trust health services provided by their doctors.”

Others think it is not a matter of trust, but a matter of available choices. Many villagers do not really have choices due to limited availability of higher-level health resources (e.g., tertiary hospitals) in rural areas and a lack of resources for travelling. They then will have to turn to whatever is reachable, namely the primary care service provided by general practitioners.

“There is only one township/village hospital at the place. “I will go to this doctor” with or without family doctor system”. “In this case, family doctor is just a formal title. No matter what this doctor is called (family doctor, or whatever), I still have to go to him/her. However, urban residents have so many medical resources, non-limited choice.”

#### 3.2.4 Nurses are lower valued

In comparison to other countries, nurses are generally undervalued in China according to our respondents. They are regarded as “doctor's assistants” and not seen as independent professionals. They hardly will be granted responsibility to innovate. Moreover, people can be resistant to nurse led health services, where doctors are not explicitly involved or involved in a limited way. However, many innovations from abroad, especially in social care are dominantly led and carried out by nurses, such as homecare for the elderly. This may inhibit the implementation of such innovations in China.

“I think that is also kind of culture. And in the hierarchical organization, the doctor is the boss, everybody listens to the doctor. Doctors take responsibilities in many ways, which leads to less responsibilities for the nurse. I think that makes a profession of a nurse or caregiver in China less effective, because they only listen to what the doctor said, and to do it. They are more like an assistant to a doctor, not being valued as professionals.”

“But their first reaction is: you (community caregiver) are not capable of providing health services; and you can come to me to wash and bathe me, change my clothes; But for health services, I would like to go to the tertiary hospitals.”

### 3.3 Values related to organizational dynamics

Within this category, four specific values and norms were discussed by our respondents namely. The values guanxi and preferring group-work over individual work relate to Hofstede's collectivism dimension. The other two values relate to hierarchy and to eagerness to learn from other cultures.

#### 3.3.1 Hierarchy

Many respondents mentioned that health organizations in China have a strict hierarchy between superiors and subordinators: between managers and employees; between senior doctors and junior doctors; between doctors and nurses. This manifests itself in specific expectations for both superiors and subordinates. While superiors are expected to make decisions, subordinators are expected to obey instructions and “overcome all difficulties” to complete assigned tasks. As a consequence, decision making can often go fast according to our respondents, which may facilitate adoption of innovations, and the implementation of technological innovations. At the same time, hierarchical relationship may be a barrier for innovations that require delegation of power to lower levels.

“There has also been a lot of resistance. Because Chinese have no precedent example in this regard. So they still believe in the traditional way of management. It’s hard for them to believe that if you decentralize the power to nurses and caregivers, they won’t make a mess of it.”

“Because Chinese are very comfortable with the way: the leader let me do A, then I will do A. It would be bad for me if I do something that is beyond the leader’s order.”

“The only solution to this problem in China is unconditional obedience. To be honest, you have to overcome difficulties yourself. That’s the way of life in China in general. You live in this environment. Just like if we went to Austria, which is a new environment, it is impossible for the environment to adapt to me. Only I can adapt to the environment.”

One Chinese respondent gave an example of how hierarchy in the clinical department influences the final decision on the surgery method for patients with gastrointestinal diseases:

“Older doctors do not implement minimally invasive surgery which is an innovation beneficial to patients’ faster recovery. Our superior doctors (about 40–50 years old), still follow the traditional surgery method in textbooks.”

“I think the hierarchy is too strict. Superior doctors need to be respected, and their instructions need to be obeyed. If junior doctors don’t listen to their superior’s opinions and then something goes wrong, it would become a personal problem.”

“So in general, if the idea of implementing the innovation comes from superior doctors, it normally will be put into practice. Different opinions from others (especially from junior doctors) may not ultimately influence the final decision.”

#### 3.3.2 Collectivism

The value and norm of collectivism indicates that people's self-image is “We” instead of “I” and personal relationships prioritize over tasks (4). Two values discussed in the interviews are related to China's high degree of collectivism, namely guanxi and group-working welcomed over individual working by nurses.

##### 3.3.2.1 Guanxi (relationship)

In Chinese organizations, guanxi is a curial system of beliefs, which is grounded in mutual commitment, reciprocity and trust (26). Preferential treatments and convenience are mutually provided and enjoyed by partners in an established guanxi. The establishment of guanxi can be achieved by social activities outside the working environment, such as dinners. According to our respondents the emphasis on guanxi in China can positively and negatively impact the implementation of innovations from abroad.

“So you have to understand that, because you will become, when you start collaborations and you do it in a good way, and you become a partner of that Guanxi; and this network is about trust. And your network in a way, yes, you are part of the family. You become a part of family if you want or don’t want, you will. That has some responsibilities, because when they ask you to do something, you do that and they will do it for you too, always.”

Both Chinese and Dutch respondents mentioned that established social ties between foreign experts and health organization managers, and among colleagues within the organization are beneficial to the implementation of foreign innovations.

“But overall, I think if you work in a certain hospital, and you will gradually find a way to get along with people there and once you have a good relationship with your colleagues, you are more likely to achieve the innovation.”

Meanwhile some Dutch respondents mentioned that the need to understand and establish Guanxi can also inhibit innovations, especially when multiple organizations (often including foreign organizations) need to be involved.

“Often organizations will start the talk, but that’s impossible in China. You also have to know more about what is Guanxi, the network. How does that work. It is not like how it is working in a Western world, in the Netherlands. But if you don’t understand, it will also not be a success.”

##### 3.3.2.2 Group-working welcomed over individual working by nurses

Several of our Dutch respondents mentioned that nurses in China seem to prefer training and working in a group (in a hospital) to working individually (in the community). This might be due to psychological safety, self-perceived low status, and the underappreciation of primary care. It might also relate to general collectivist values and correspondingly being trained to work collectively rather than independently. However, innovations from the West do increasingly require nurses to work independently in homecare settings. Such innovations may thus encounter implementation challenges.

“You see a lot of nurses can work in the hospital, and the hospital is kind of a safe environment for a nurse. But you don’t see skilled nurses in elderly care. You don’t have the security, colleagues in the hospital, and you have to do it on your own.”

“Also the training is a little bit different between a hospital nurse and a home care nurse. Home care is more individualistic, you must do it your own. You also take a little bit entrepreneurship, so thinking, ‘what can I do, if I don’t have help.’ It is more on your own. And that asks for a different education also.”

##### 3.3.2.3 Eagerness to learn from other cultures

Some respondents mentioned that Chinese health organizations are enthusiastic to learn from and exchange experiences with overseas counterparts. As written in *“Confucian Analects”, “when three are walking together, I am sure to find teachers among them”* (27). Traditionally the Chinese are stimulated to be modest, to recognize one's weaknesses and always look for opportunities to learn from others. Since China's health reform initiated in 2009, China decided to improve its health system by innovating and learning from others (28). This eagerness to learn from others facilitates the implementation of international practices in China.

“I think, what I noticed in China… I think they are very eager to learn from everywhere. They have contact, the social welfare home, they have contacts with Denmark, with Japan, Philippine, Canada..Netherlands. All different systems.”

## 4 Discussion

The purpose of this study was to identify which cultural values and norms influence the adoption and implementation of foreign innovations in health service delivery in the Chinese context and how. We identified 12 values and norms, subdivided in three categories namely (1) *health and care*, referring to values and norms influencing people's expectations and preferences on health and care; (2) *health services and professionals*, referring to values and norms influencing peoples' perceptions towards and choices on health services, professionals, and healthcare settings; and (3) *organizational dynamics*, referring to values and norms influencing peoples' behaviors, learning styles in a workplace and patterns of interactions between people within and outside the organization.

Our respondents especially identified values and norms which act as potential barriers to the adoption and implementation of innovations. Additionally, some of the identified factors are a facilitator or barrier depending on the circumstances or the views of our respondents, and one is purely a facilitator. Supplementary Table S2 summarizes the influence of cultural values and norms on the adoption and implementation of foreign innovations in health service delivery in the Chinese context. This table illustrates the potential barriers (e.g., the body must be treated with respect), facilitators (e.g., eagerness to learn from other cultures), and context-dependent factors influencing the adoption of innovations (e.g., filial piety, longevity is valued over intangibles).

National values and norms are found to influence the adoption and implementation of innovations (16). Supplementary Table S1 summarizes previous evidence on how national cultures affect the adoption and implementation of innovations in the Chinese context. For instance, high scores on the dimensions power distance, collectivism, and masculinity have been reported to positively impact adoption and implementation of innovations, while a negative impact is reported for high scores regarding uncertainty avoidance, and long-term orientation.

Many of the values we've identified seem to match the national values for China as identified by studies using Hofstede's model, with some exceptions (6). However, we found that the impact these values have on adoption and implementation of innovations in health service delivery varies. We also identified several important values which were not mentioned by Hofstede (29). It seems that using national culture as a lens is insufficient to describe cultural influences of implementing innovations in China. Greenhalgh et al. (16), identified different levels on which norms and values may influence implementation of innovations (e.g., system readiness for innovation). They describe how values may differ between regions, sectors and stakeholders and how these values may also clash. Furthermore, the innovation itself may have certain characteristics that do or do not match with specific values (e.g., system-innovation fit) (16).

Previous studies have shown that collectivism positively influences the implementation of innovations, because “group culture” which encourages working cooperatively and closely is beneficial to coordinating budgets, schedules, human resources for implementing innovations (relating to “guanxi”, “group working”) (6, 30). We found that when guanxi is established with foreign partners or experts, this strongly facilitates diffusion of innovations. However, our evidence also suggests that Chinese collectivism may present challenges for innovations that require individualistic and independent working styles, particularly in contexts where collaboration and group cohesion are highly valued. While collectivism can foster strong teamwork and shared responsibility, it may limit the acceptance of innovations (e.g., homecare provided by individual nurses) that prioritize autonomy or individual decision-making, especially in settings where these values conflict with traditional cultural norms. This dynamic may vary depending on the specific context, such as the type of innovation or the sector in which it is introduced.

Similarly, masculinity was also found in previous studies to have a positive impact on innovation implementation (6, 30), because greater emphasis on masculine qualities (including purposes, performance, achievement) than on feminine qualities (human relations, communication, team spirit) contributes to formalizing tasks and participants' roles in implementing innovations. Our respondents also mention in this context the decisiveness of Chinese managers, which speeds up innovation. However, our findings indicate that Chinese masculine values may also inhibit innovations. Especially when it relates to new service concept innovations in elderly and long-term care which prioritizes feminine qualities (e.g., emphasis on people, life quality, empathy).

Furthermore, previous studies have found that high power distance has a negative impact on innovation adoption, due to less initiative taken by subordinators and unrealistic expectations on supervisors' abilities to identify all operational problems (5). However, our findings suggest that in the Chinese context subordinates have much experience with top-down decisions and dealing with local adaptations within health organizations. Especially, when innovations are less complex and clearly structured, top-down implementation can stimulate implementation of innovations by speeding up decision making processes,.

Previous studies suggest that the Chinese have a relative open attitude towards risk taking, because of their low level of uncertainty avoidance (6, 30). However, we found that within the health care sector professionals are often reluctant to take risks (16). This seems to relate to the fact that within the Chinese health care sector different stakeholders attach different values to services and innovations (see also Greenhalgh et al) (16). While health professionals in China regard health service provision as a public service; a commitment to societal well-being, Chinese patients tend to regard health services as economic transactions and expect value for money. Conflicts may therefore easily arise if treatment outcomes are not satisfying. At the same time, much value is placed in the Chinese system on tangibles such as facilities and equipment, over intangibles such as communication and empathy. Under these circumstances, professionals tend to prioritize their safety and professional reputation and avoid taking the risk of innovations, especially when of intangible nature.

Studies using the Hofstede model often suggest that the Chinese are long-term and future-oriented, which stimulates innovations (30). In cultures with a long-term orientation, there is a greater focus on perseverance, thrift, and adapting to future challenges. This often manifests in health service delivery through the prioritization of preventative measures and investments in sustainable healthcare systems, such as China's increasing focus on integrating traditional medicine with modern practices for long-term well-being. Our findings, however, echo the studies showing that the Chinese culture can also be short-term and past-oriented (6, 29, 31). Our respondents for example mention the importance placed in upholding traditional values such as values related to Traditional Chinese Medicine and filial piety. But they also mention important differences between regions and how traditional values are reinterpreted to fit in modern society. The duty of a child to care for parents (“filial piety”) is a shared value in Chinese society. The Hakka (literally mean “guest families”), as a subgroup whose ancestral homes are in different provinces, tend to follow the old tradition relating to filial piety in which children must provide personal services to their parents and thereby refusing innovations such as community-based services for the elderly (32). Meanwhile, in developed cities these traditions have been reinterpreted to fit modern family constellations which facilitate the outsourcing of duty of care for parents and service innovations for the elderly (33).

Our study provides new perspectives on cultural influences on adoption and implementation of foreign innovations in health service delivery in China. This evidence can serve to advance further research and better inform policy and practice on cultural barriers and facilitators. First, it is of great importance to consider compatibility between innovations and existing Chinese values and norms. Moreover, it is important to recognize that values and norms may vary between sectors and between stakeholders (mainly health providers and patients). It should be recognized that China is a large country with diverse sub-cultures. Considerable differences exist, for instance between Han- and Minorities- dominated regions.

To support foreign innovation adoption policy makers and professionals should therefore first try to understand the compatibility between the innovation and the context specific values and norms. Pilot programs can help to test this compatibility and identify the needs for adaptation of both the innovation and the implementation context. An iterative process in which the innovation and the context are adapted stepwise maybe required. Additionally, collaborative learning between local providers and cross-cultural experts may help bridge cultural gaps and facilitate smoother adoption of innovations.

## 5 Limitations

There are several limitations of this study. First, the number of respondents is limited, and most Chinese respondents are mainly from central and eastern China. Considering regional differences in culture, it is possible that some other relevant values and norms, for instance in western regions of China have not been identified. Second, foreign respondents in our study are exclusively from the Netherlands, but foreign innovations introduced are not limited to Dutch innovations. Although unique and distinct insights may be provided due to obvious cultural differences between the Netherland and China, some values and norms may be found more relevant than others for respondents with different cultural backgrounds. For instance, Dutch culture has a more open thanatology, therefore innovations focusing on quality of life may be more frequently used as an example by Dutch respondents. Finally, the selection method of respondents may have introduced several forms of bias. Snowballing introduces the risk of selecting respondents with similar experiences and perspectives as they may be well connected. By asking the Chinese Ministery to help with connecting to respondents, we may have missed respondents whose views differ from the ministerial views.

## 6 Conclusion

Cultural values and norms related to health and care, health services and health professionals, and organizational dynamics are identified in this study as facilitators and/or barriers for the adoption and implementation of foreign innovations in health service delivery in the Chinese context. Especially innovations in primary and elderly care that are nurse-led and focus on new service concepts emphasizing personalized care, experience cultural barriers in China. At the same time, more structural and technical innovations especially in a hospital setting are strongly facilitated by Chinese cultural characteristics. While national culture has a significant impact on foreign innovations in China, the interaction with the cultural context at meso- and micro- level and the compatibility of existing values with characteristics of the innovation itself should be taken into consideration.

## Data Availability

The raw data supporting the conclusions of this article is not readily available due to the confidentiality of respondents. Further queries can be directed to the corresponding author.
