# Supplementary material for: The role of cultural values and norms in the adoption and implementation of foreign innovations in health service delivery in China
Source: Front Health Serv. 2025 Jul 18;5:1401641. doi: 10.3389/frhs.2025.1401641 (PMC12313577; doi:10.3389/frhs.2025.1401641)
Supplement: Supplementary file 1 [file Table1.docx]

Table 1. The effect of Hofstede’s five dimensions of national cultures on the adoption and implementation of innovations

| **Dimensions** ^4^ | **Definitions (Hofstede insights)** ^4^ | **National cultural characteristics (China)** ^4^ | **Effects on the adoption and implementation of innovations in the Chinese context**^5,6,27^ |
| --- | --- | --- | --- |
| Power distance index (PDI) | ‘*The extent to which the less powerful members of institutions and organizations within a country expect and accept that power is distributed unequally’* | PDI (China): 88  PDI (Netherlands): 38  High power distance  Chinese organizations are characterized by centralized decisions, authority, formal rules, and constrained by hierarchy. | Positively contributes to the implementation of innovations because authority, rules and hierarchy are helpful to:   - Coordinating the institutionalization of innovation, the mechanisms of communication and coordination, and time schedules for innovations - Overcoming resistance to innovations to be executed, and changes needed among staff and departments to bring innovations to realization   Negatively contributes to the adoption of innovations, because:   - Top management is not always able to identify operational problems, and to suggest the introduction of innovations to solve relevant problems - Subordinators may take less initiatives to discuss and consider innovations, and wait for the top management to take initiative |
| Individualism index (IDV) | ‘*The degree of interdependence a society maintains among its members’.* | IDV(China): 20  IDV(Netherlands):100  Collectivism  People’s self-image is “We” instead of “I” and personal relationships prioritize over tasks. “Group culture” creates a sense of belonging. | Positively contributes to the implementation of innovations, because:   - Collectivism is helpful to working cooperatively and closely with one another, which are required to ensure that budgets, schedules, human resources, and objectives are met.   Negatively contributes to the adoption of innovations, because:   - Collectivistic organizations act conforms to the norms of group, and are characterized by collective decisions, which may lead to the delay in the innovation adoption. |
| Masculinity index (MAS) | ‘*The fundamental issue here is what motivates people, wanting to be the best (Masculine) or liking what you do (Feminine)’* | MAS (China): 66  MAS (Netherlands): 14  Masculinity  Chinese society is masculine and put greater values on achievement, money, purposes, performance, tasks; while less emphasis is put on people, life quality, helping others. | Positively contributes to the adoption and implementation of innovations, because:   - Purposefulness in masculine society is critical to successfully adopt innovations. - Formalization of tasks and roles enable all participants to clearly know what should be done by whom and how it is to be done. |
| Uncertainty avoidance index (UAI) | ‘*The extent to which the members of a culture feel threatened by ambiguous or unknown situations and have created beliefs and institutions that try to avoid these*’. | UAI (China): 30  UAI (Netherlands): 53  Low uncertainty avoidance  China with low uncertainty avoidance show characteristics such as courage and curiosity to innovations. Less risk-averse attitudes imply the willingness to take necessary tasks. | Positively contributes to the adoption of innovations, because:   - Risk taking is a key value that promotes innovation.   Negatively contributes to the implementation of innovations, because:   - Insufficient planning due to high UAI can not reduce the possibility of errors by managing the nature and extent of plausible events. |
| Long-term orientation (LTO) | *‘How every society has to maintain some links with its own past while dealing with the challenges of the present and future’* | LTO (China): 87  LTO (Netherlands): 67  Long-term orientation  Although China is regarded as a country with long-term orientation in Hofstede model, Qin^6^ argued that current Chinese society is actually short-term orientation in reality.  People have static mentality focused on the past and present, such as reciprocation, “face”, and tradition. | Negatively contributes to the adoption and implementation of innovations, because   - Maintaining time-honored traditions are not beneficial to innovations. - The coordination of resources to improve the effectiveness of implementing innovations is challenged by putting emphasis on short-term benefits. |
