# Supplementary material for: The role of cultural values and norms in the adoption and implementation of foreign innovations in health service delivery in China
Source: Front Health Serv. 2025 Jul 18;5:1401641. doi: 10.3389/frhs.2025.1401641 (PMC12313577; doi:10.3389/frhs.2025.1401641)
Supplement: Supplementary file 2 [file Table2.docx]

Table 2: Values and norms influencing the adoption and implementation of foreign innovations in health service delivery in the Chinese context

| **Category 1: Values and norms related to health and care** | | |
| --- | --- | --- |
| **Values and norms** | **Facilitator/Positive impact** | **Barrier/ Negative impact** |
| Expectations of familial caregiving | X:  In developed cities such as Shanghai | X:  In Chaoshan region dominated by Hakka culture |
| The body must be treated with respect |  | X:  Evidenced-based medicine and practices |
| The values of Traditional Chinese Medicine | X:  Innovations related to integrative medicine, according to Chinese respondents | X:  Innovations related to evidence-based practices, according to Dutch respondents |
| Longevity is valued over quality of life | X：  Technological innovations | X:  Innovations in hospice care |
| **Category 2: Values and norms related to health services and professionals** | | |
| **Values and norms** | **Facilitator/Positive impact** | **Barrier/ Negative impact** |
| Tangibles valued over intangibles | X:  Technological innovations | X:  Service concept innovations (e.g., personalized care) |
| Health care as public service vs. economic transaction |  | X:  Innovations engaging health professionals (e.g., new surgery methods) |
| Undervalued and distrusted health professionals and services in primary care |  | X:  Innovations in primary care (e.g., family doctor services) |
| Nurses are lower valued |  | X:  Innovations with nurses’ dominant participation (e.g., homecare for the elderly) |
| **Category 3: Values and norms related to organizational dynamics** | | |
| **Values and norms** | **Facilitator/Positive impact** | **Barrier/ Negative impact** |
| Hierarchy | X:  Technological innovations | X:  Managerial innovations (e.g., flat management) |
| Guanxi | X:  Established guanxi has a facilitating role | X:  Guanxi is difficult to establish if multiple organizations need to be involved |
| Group-working welcomed over individual working by nurses |  | X:  Innovations requiring nurses’ independence (e.g., homecare for the elderly) |
| Eagerness to learn from other cultures | X:  Purely a facilitating role in general |  |
